# Toxicity Associated with Stavudine Dose Reduction from 40 to 30 mg in First-Line Antiretroviral Therapy

**DOI:** 10.1371/journal.pone.0028112

**Published:** 2011-11-21

**Authors:** Mar Pujades-Rodríguez, Emmanuelle Dantony, Loretxu Pinoges, René Ecochard, Jean-François Etard, Esther Carrillo-Casas, Elisabeth Szumilin, for the AIDS Working Group of Médecins Sans Frontières

**Affiliations:** 1 Clinical Research Department, Epicentre, Paris, France; 2 Service de Biostatistique, Hospices Civils de Lyon, Lyon, France; 3 Unité Mixte de Recherche 5558, Centre National de Recherche Scientifique, Villeurbanne, France; 4 Service de Biostatistique, Université de Lyon, Lyon, France; 5 Unité Mixte Internationale 233, Institut de Recherche pour le Dévelopment, Montpellier, France; 6 Medical Department, MSF AIDS Working Group, Amsterdam, Holland; 7 Medical Department, MSF AIDS Working Group, Paris, France; Institute of Infectious Diseases and Molecular Medicine, South Africa

## Abstract

**Background:**

To compare the incidence and timing of toxicity associated with the use of a reduced dose of stavudine from 40 to 30 mg in first-line antiretroviral therapy (ART) for HIV treatment and to investigate associated risk factors.

**Methods:**

Multicohort study including 23 HIV programs in resource-limited countries. Adults enrolled between January 2005 and December 2009. Four-year rates of all-cause and stavudine-specific toxicity were estimated. Multilevel mixed-effect Poisson and accelerated failure models were used to investigate factors associated with toxicity and timing of diagnosis.

**Findings:**

A total of 48,785 patients contributed 62,505 person-years of follow-up. Rate of all-cause toxicity was 7.80 (95%CI 7.59–8.03) per 100 person-years, but varied greatly across sites (range 0.41–21.76). Patients treated with stavudine 40 mg had higher rates of toxicity (adjusted rate ratio [aRR] 1.18, 95%CI 1.06–1.30 during the first year of ART; and 1.51, 95%CI 1.32–1.71 during the second year). Women, older age, initial advanced clinical stage, and low CD4 count were associated with increased toxicity rate ratios. Timing of lipodystrophy and peripheral neuropathy diagnosis were 12% and 13% shorter, respectively, in patients treated with stavudine 40 mg than in those receiving 30 mg stavudine dose (*P* = 0.03 and 0.07, respectively).

**Insterpretation:**

Higher rates of drug-related toxicity were reported in patients receiving stavudine 40 mg compared with 30 mg, and the time to toxicity diagnosis was shorter in patients treated with the higher dose. Higher rates of toxicity were observed during the first two years of ART.

## Introduction

Stavudine is a dideoxynucleoside agent active against HIV. It is widely used for HIV treatment in resource-limited countries due to its low cost, availability in double and triple generic fixed-dose combinations of antiretroviral (ARV) drugs, favorable early tolerability, convenient drug administration, and minimal requirements for laboratory monitoring.

The main adverse effects associated with stavudine use are symmetrical peripheral neuropathy and lipodystrophy. These are dose-related [1]–[5] and the neuropathy is clinically indistinguishable from primary HIV-related distal polyneuropathy [6], [7]. Based on findings from a systematic review of clinical trials showing equivalent antiviral efficacy and some evidence of lower rates of stavudine-related toxicity [8], in 2007 the World Health Organization (WHO) recommended decreasing the dose of stavudine-containing first-line ARV therapy (ART) from 40 mg to 30 mg twice daily for adults irrespective of body weight [9]. The effectiveness of this strategy to reduce or delay the development of toxicity has not been evaluated.

Although the WHO has recommended replacing stavudine-based regimens with less toxic combinations in first-line ART, stavudine-containing therapies continue to be prescribed to 57% of newly treated patients in low- and middle-income countries [10] because of lack of affordable alternative regimens [11]. Because phasing out of stavudine is not likely to be adopted in the coming years by several countries, WHO is currently assessing whether further reducing stavudine dosage to 20 mg BID could achieve and maintain adequate virologic efficacy while reducing the incidence and intensity of mitochondrial toxicity.

To guide decision-makers and ensure dose optimization and appropriate use of effective ARV regimens, it is important to understand the safety profile of alternative dosing of stavudine and the factors that contribute to the development of adverse reactions. In this analysis we compared toxicity associated with stavudine dose reduction from 40 mg to 30 mg BID for the treatment of HIV infection and investigated risk factors for and timing of toxicity diagnosis in 23 HIV programs supported by Médecins Sans Frontières (MSF).

## Materials and Methods

### Cohort Selection and Study Population

We analyzed individual patient data from all active MSF-supported HIV programs using the electronic FUCHIA monitoring software (Epicentre, Paris, France), with a loss to follow-up rate <20% at one year since ART start and with at least 50 patients meeting the study selection criteria. All patients aged ≥15 years old with no prior history of ART use and who started on a stavudine-containing first-line ARV regimen between 1 January 2005 (date of start of collection of ARV toxicity information) and 31^st^ December 2009 were included. Regimens were administered in the form of a fixed-dose combination of ARVs. Because onset of stavudine-related toxicity has been reported to happen between weeks 5 and 46 of treatment (22 weeks when 0.5 mg/kg/dose BID is used) [2], the primary analysis used only information from patients who had received a stavudine-based regimen for ≥3 months.

### Data Collection and Definitions

At each consultation or hospitalization individual patient data were prospectively collected using standardized forms and entered into FUCHIA. Data collected included sex, age, date and type of toxicity, dates of appointment and clinical visits, CD4 cell count measurements, and date of ART start. No patient identifiers were kept in the datasets analyzed.

The primary outcome was the first recorded toxicity diagnosis, whether or not it led to a change of ART regimen (all-cause toxicity). The secondary outcomes were specific stavudine-related toxicity (first recorded diagnosis of lipodystrophy, polyneuropathy, lactic acidosis, and/or pancreatitis), timing of toxicity diagnosis, and toxicity leading to stavudine interruption. All toxicity events were included regardless of severity grading, and definitions of toxicity were based on the grading scale from the Division of AIDS, National Institute of Allergy and Infectious Diseases, version 1.0 December 2004.

### Ethics

In agreement with health ministries, data were prospectively collected. No patient identifiers were kept in the data sets analysed, and proposals for analysis were approved by the International Ethics Review Board of MSF. As advised by the National Commission of Informatics and Liberties, given the context in which MSF works, information for patients about the data collection system in the health facility and its use is provided to patients verbally at program inclusion instead of obtaining written informed consent.

### Statistical Analysis

Patient follow-up started at the beginning of ART and was right-censored at the earlier of the following events: date of last clinical visit with prescription of a stavudine-containing regimen, change in stavudine dose, death, transfer, toxicity diagnosis, or 4 years. For each patient, total stavudine exposure was calculated multiplying the total number of days receiving a stavudine-containing regimen by the daily dose of stavudine administered (in mg). Kaplan-Meier methods were used to describe 4-year cumulative toxicity. Rates of all-cause and specific toxicity (whether or not it led to stavudine interruption) and rates of toxicity leading to stavudine interruption with Poisson exact 95% confidence intervals (CI) were estimated at 6 months by stavudine dose group.

To investigate the association between toxicity and stavudine exposure, and associations with other potential risk factors, multilevel mixed-effect Poisson models with random effects at cohort level were used to account for heterogeneity between programs. This was done by including stavudine dose (30 and 40 mg), duration of stavudine exposure in years, and an interaction term between these two variables in the models. Factors considered in the analysis were: sex; age (centered and introduced as a continuous variable), CD4 cell count (<50, 50–99, 100–199, ≥200, and missing cells/µl), body mass index (BMI, <16, 16–18.49, 18.50–24.99, ≥25, and missing kg/m^2^), active tuberculosis disease, and clinical WHO stage (1 or 2, 3, 4, and missing) at ART start; period of toxicity diagnosis (yearly from 2005 to 2009); and adherence (>95% and ≤95% incident missed appointments) [12]. Analyses restricted to the subgroup of patients with complete data (BMI, clinical stage, and CD4 cell counts) were also performed.

Because the pathophysiological mechanisms responsible for the development of toxicity are unclear and might vary for different types of toxicity, accelerated failure time models were used to investigate time to first diagnosed episode of neuropathy and lipodystrophy, the two main types of stavudine-related toxicities diagnosed. Delay in diagnosis was modeled using a log-normal distribution for neuropathy and a Weibull distribution for lipodystrophy. The choice of models was based on the study of Cox-Snell and deviance residuals obtained when using different distributions. Factors considered in the analyses were the same as listed before, except for the exclusion of the period of toxicity diagnosis and duration of drug exposure and the inclusion of the year of ART start. In addition, the variable cohort was included as a fixed effect in the model for neuropathy, and type of location (rural, urban, and semi-urban) in the model for lipodystrophy, given the smaller number of recorded events for this type of toxicity. In sensitivity analyses, timing of toxicity was studied when episodes of toxicity recorded within 3 months of ART use were also included (1 lipodystrophy and 731 peripheral neuropathies). This was done because HIV-related distal symmetric polyneuropathy is more frequently diagnosed in patients with advanced HIV disease [13]–[15]; the two types of peripheral neuropathy are clinically similar, often overlap, and are likely to result in additive or synergistic effects; and because patients in our programs started ART in late HIV disease.

All analyses were performed in Stata 11 (StataCorp, College Station, TX, US).

## Results

A total of 62,505 person-years of follow-up from 48,785 patients treated in 23 HIV programs were analyzed (Table S1; Figure S1), 10,960 from patients who received stavudine 40 mg and 51,544 from patients treated with stavudine 30 mg. Sixty-seven per cent were women and median age at ART initiation was 36 years (Table 1). Median cumulative exposure to stavudine was 36,192 mg for patients treated with stavudine 40 mg and 22,709 mg for those who received stavudine 30 mg. Regardless of stavudine dose, the majority of patients timely attended clinical visits during follow-up (99.5% had an adherence index of ≥95%).

**Table 1:** Characteristics of ART patients by stavudine dose group.

	d4T 40 mg	d4T 30 mg	Total
	N = 7,813	N = 40,972	N = 48,785
**Median age, years [IQR]**	37.3 [31.5–44.1]	35.2 [29.7–42.8]	35.6 [30.0–43.0]
**Women (%)**	4303 (55.1)	28,176 (68.8)	32,479 (66.6)
**Year of ART start (%)**			
2005	2592 (33.2)	7000 (17.1)	9592 (19.7)
2006	3134 (40.1)	9132 (22.3)	12,266 (25.1)
2007–2009	2087 (26.7)	24,840 (60.6)	26,927 (55.2)
**Clinical stage (%)**			
1/2	2713 (35.8)	10,517 (26.5)	13,230 (28.0)
3	3558 (46.9)	19,976 (50.4)	23,534 (49.8)
4	1316 (17.4)	9156 (23.1)	10,472 (22.2)
**TB diagnosis at ART start (%)**	629 (8.1)	4828 (11.8)	5457 (11.2)
**Median weight, kg [IQR]**	66.0 [62.0–71.5]	51.0 [46.0–56.0]	53.0 [47.0–60.0]
**Median BMI, kg/m^2^ [IQR]**	23.7 [21.7–26.2]	19.5 [17.7–21.4]	20.1 [18.1–22.3]
**CD4 cell count, cells/**µ**L**			
Median [IQR]	144 [80–195]	132 [64–197]	135 [67–197]
<50	914 (14.7)	6309 (19.7)	7223 (18.9)
50 – 99	1088 (17.4)	5817 (18.2)	6905 (18.0)
100 – 199	2842 (45.5)	12,213 (38.1)	15,055 (39.3)
≥200	1397 (22.4)	7710 (24.1)	9107 (23.8)
**Median cumulative stavudine exposure, mg [IQR]**	38,952 [23,340–58,191]	24,069 [13,247–38,972]	26,080 [14,134–42,638]
**Adherence index** 1			
≥95%	7761 (99.5)	40,672 (99.5)	48,433 (99.5)
<95%	43 (0.5)	222 (0.5)	265 (0.5)

Abbreviations: ART, combined antiretroviral therapy; BMI, body mass index; d4T, stavudine; IQR, interquartile range; TB, tuberculosis.

Note: 1549 with missing clinical stage at ART start; 372 with missing weight at ART start; 7768 with missing BMI at ART start; 10,495 with missing CD4 cell count at ART start.

1 Adherence index is calculated as the incidence of appointment attendance with no delay.

### All-Cause Toxicity

All-cause toxicity during the first 4 years of ART use was diagnosed in 4878 (10%) patients after a median of 13 months of ART start (Table 2). Of these, 2234 (45.8%) patients interrupted stavudine use. Overall all-cause toxicity rate was 7.80 per 100 person-years (95%CI 7.59–8.03); but rates varied greatly across sites, from 0.41 to 21.76 per 100 person-years. Cumulative probability of toxicity was higher for patients who received high-dose stavudine (Figure S2A). Four-year toxicity rates were 9.63 per 100 person-years (95%CI 9.07–10.23) in patients who received stavudine 40 mg and 7.41 (95%CI 7.18–7.65) in those treated with stavudine 30 mg.

**Table 2:** Rates of toxicity per stavudine dosage group and time of stavudine exposure.

	No. of events(%)	Months on ART to event [IQR]	Toxicity rate/100 PY(95% CI)	[3-6] month toxicity rate/100 PY (95% CI)	[6-12] month toxicity rate/100 PY(95% CI)	[12-18] month toxicity rate/100 PY(95% CI)	[18-24] month toxicity rate/100 PY(95% CI)	[24-48] month toxicity rate/100 PY(95% CI)
**ALL-CAUSE TOXICITY**								
**Stavudine 40mg**	1056(13.5)	15.1[9.0-23.1]	9.63(9.07-10.23)	6.01(5.28-6.84)n = 227	13.22(11.97-14.60)n = 387	13.33(11.82-15.04)n = 264	9.56(7.96-11.47)n = 115	5.88(4.59-7.52)n = 63
Only SA	1044(13.5)	15.1[9.0-23.2]	9.57(9.01-10.17)	5.94(5.21-6.78)n = 223	13.12(11.87-14.50)n = 382	13.27(11.76-14.98)n = 262	9.49(7.60-11.40)n = 114	5.88(4.59-7.53)n = 63
**Stavudine 30mg**	3822(9.3)	12.6[6.9-20.6]	7.41(7.18-7.65)	5.70(5.37-6.04) n = 1109	10.20(9.67-10.76) n = 1361	7.87(7.29-8.49)n = 669	6.08(5.43-6.80)n = 305	7.24(6.55-8.01)n = 378
Only SA	3,682(9.2)	12.6[6.9-20.7]	7.31(7.08-7.55)	5.74(5.41-6.09) n = 1088	10.16(9.62-10.72) n = 1317	7.42(6.86-8.03)n = 616	5.77(5.14-6.48)n = 286	7.22(6.53-7.99)n = 375
**Total**	4878(10.0)	13.0[7.2-21.1]	7.80(7.59-8.03)	5.75(5.45-6.07) n = 1,336	10.74(10.25-11.26) n = 1748	8.90(8.35-9.49)n = 933	6.75(6.13-7.43)n = 420	7.01(6.39-7.70)n = 441
Total SA	4726(9.9)	13.0[7.1-21.2]	7.71(7.50-7.94)	5.77(5.47-6.09) n = 1311	10.70(10.21-11.22) n = 1699	8.54(8.00-9.13)n = 878	6.50(5.89-7.17)n = 400	6.99(6.37-7.68)n = 438
**SPECIFIC TOXICITY**								
**Stavudine 40mg**	882(11.3)	15.2[9.1-23.3]	8.00(7.49-8.55)	5.05(4.39-5.82)n = 191	11.37(10.21-12.65)n = 334	10.98(9.61-12.53)n = 219	7.47(6.09-9.18)n = 91	4.30(3.23-5.72)n = 47
Only SA	871(11.2)	15.2[9.1-23.4]	7.94(7.43-8.48)	5.01(4.34-5.78)n = 188	11.26(10.11-12.55)n = 329	10.91(9.55-12.47)n = 217	7.40(6.02-9.10)n = 90	4.30(3.23-5.72)n = 47
**Stavudine 30mg**	3346(8.2)	12.7[7.0-20.7]	6.46(6.25-6.68)	4.93(4.62-5.25)n = 959	8.86(8.37-9.37)n = 1186	7.02(6.48-7.61)n = 601	5.39(4.79-6.07)n = 273	6.17(5.53-6.87)n = 327
Only SA	3,219(8.1)	12.6[6.9-20.9]	6.36(6.14-6.58)	4.95(4.64-5.28)n = 939	8.81(8.31-9.33)n = 1146	6.63(6.10-7.20)n = 554	5.10(4.51-5.76)n = 255	6.16(5.52-6.86)n = 325
**Total**	4228(8.7)	13.1[7.2-21.2]	6.73(6.53-6.94)	4.95(4.6-5.24)n = 1150	9.31(8.85-9.79)n = 1520	7.77(7.26-8.32)n = 820	5.79(5.23-6.42)n = 364	5.85(5.28-6.47)n = 374
Total SA	4090(8.6)	13.1[7.2-21.4]	6.64(6.44-6.85)	4.96(4.68-5.26) n = 1127	9.26(8.80-9.74)n = 1475	7.45(6.94-8.00)n = 771	5.55(4.99-6.17)n = 345	5.84(5.27-6.46)n = 372

Note: CI, confidence interval; IQR, interquartile range; PY, person-years of follow-up; SA, sub-Saharan Africa.

**Figure 1:**
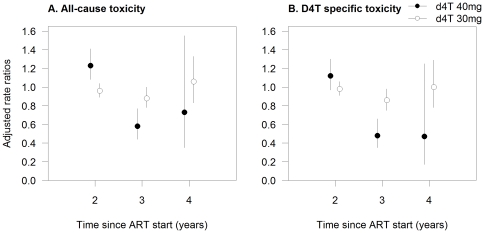
Adjusted incidence rate ratios of toxicity for duration of stavudine exposure (in years), per dosage group. Figure note: Estimates with 95% confidence intervals are derived from multilevel mixed-effect Poisson models with random effects at cohort level. Reference category is the first year of therapy exposure.

The highest rates of all-cause toxicity were seen during the 6–12 month period after ART initiation (6–18 months period for patients on stavudine 40 mg). For the two dose groups, toxicity rates during the second and fourth years of stavudine exposure were similar or higher than during the first year of treatment (adjusted rate ratios [aRR] 1.23, 95%CI 1.08–1.41; and 0.73, 95%CI 0.35–1.55, respectively, for 40 mg; and 0.96, 95%CI 0.89–1.04, and 1.06, 95%CI 0.83–1.33, respectively, for 30 mg; Figure 1A).

The rate of regimen interruption because of toxicity was 3.42 (95%CI 3.28–3.56). This was higher for patients who received regimens containing stavudine 40 mg (5.28, 95%CI 4.87–5.71, compared to 3.02, 95%CI 2.88–3.17 in patients treated with stavudine 30 mg).

### Specific Toxicity

A total of 4228 (9%) patients were diagnosed with specific stavudine toxicity during the first 4 years of ART use after a median of 13 months of ART start (Table 2). Toxicities diagnosed during the first recorded episode were 3805 neuropathy, 274 lipodystrophy, 144 lactic acidosis, and 5 pancreatitis. Of patients with recorded specific stavudine toxicity, 1753 interrupted stavudine use (1362 had peripheral neuropathy, 252 lipodystrophy, 137 lactic acidosis and 2 pancreatitis). Overall toxicity rate was 6.73 per 100 person-years (95%CI 6.53–6.94), but rates varied greatly across sites, from 0 to 20.79 per 100 person-years. As with all-cause toxicity, cumulative probability of toxicity was higher for patients who received high-dose stavudine (Figure S2B).

Four-year specific stavudine toxicity rates were higher in patients who received stavudine 40 mg than in those treated with 30 mg (8.00 compared to 6.46 per 100 person-years), and the highest values were seen during the 6–12 month period after the start of ART. Rates during the second and fourth years of stavudine exposure were not statistically different from those observed during the first year of drug use (Figure 1B). The rate of regimen interruption because of specific stavudine toxicity was 2.68 (95%CI 2.56–2.81); and it was higher for patients who received regimens containing stavudine 40 mg (4.03, 95%CI 3.68–4.42, compared to 2.39, 95%CI 2.26-2.53).

### Factors Associated with Stavudine Toxicity

**Figure 2:**
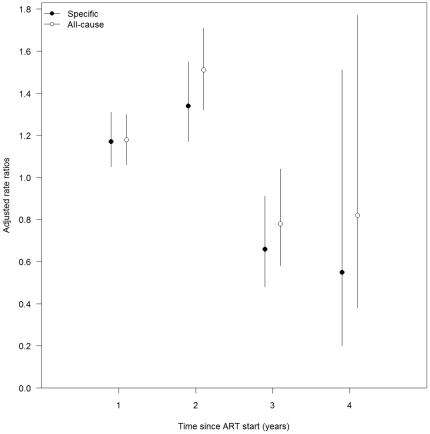
Adjusted incidence rate ratios of toxicity-related to stavudine dosage exposure (40 mg vs. 30 mg) per duration of exposure (in years) and type of toxicity with 95% confidence intervals.

During the first year of ART use, all-cause and specific stavudine toxicity were more frequently diagnosed in patients who received stavudine 40 mg than in those treated with 30 mg (aRR 1.18, 95%CI 1.06–1.30 and aRR 1.17, 95%CI 1.05–1.31, respectively; Table 3). Compared to patients treated with low-dose stavudine, those who used stavudine 40 mg had an increased risk of toxicity during the first 2 years of exposure but this association was reversed in the subsequent 2 years (Figure 2).

**Table 3:** Associations between toxicity and individual level factors.

	All-cause toxicity		Specific stavudine toxicity	
	aIRR (95% CI)	*P-value*	aIRR (95% CI)	*P-value*
**Stavudine dose**		<0.001		<0.001
30 mg	1		1	
40 mg	1.18 (1.06-1.30)		1.17 (1.05-1.31)	
**Time of drug exposure (year)**		<0.001		<0.001
1	1		1	
2	0.96 (0.89-1.04)		0.98 (0.91-1.06)	
3	0.88 (0.78-1.00)		0.86 (0.75-0.98)	
4	1.05 (0.83-1.33)		1.00 (0.77-1.29)	
**Age, per 1 year increase**	1.05 (1.04-1.05)	<0.001	1.05 (1.04-1.05)	<0.001
**Sex**		<0.001		<0.001
Men	1		1	
Women	1.18 (1.11-1.26)		1.16 (1.08-1.24)	
**Period of toxicity diagnosis**		<0.001		<0.001
2005	1		1	
2006	1.92 (1.67-2.21)		2.23 (1.89-2.62)	
2007	2.23 (1.94-2.56)		2.76 (2.36-3.24)	
2008	1.83 (1.59-2.12)		2.16 (1.83-2.54)	
2009	5.89 (4.49-7.74)		6.75 (4.92-9.27)	
**Clinical WHO stage**		0.004		0.026
1/2	1		1	
3	1.06 (0.99-1.14)		1.05 (0.97-1.14)	
4	1.12 (1.03-1.23)		1.06 (0.97-1.17)	
**Tuberculosis diagnosis at ART start**		0.005		0.012
No	1		1	
Yes	1.15 (1.04-1.26)		1.14 (1.03-1.27)	
**BMI group, kg/m^2^**		<0.001		<0.001
<16	1		1	
16.00-18.49	0.96 (0.85-1.08)		0.99 (0.87-1.13)	
18.50-24.99	0.87 (0.77-0.97)		0.88 (0.78-1.00)	
≥25	1.10 (0.95-1.29)		1.16 (0.99-1.37)	
**CD4 cell count, cells/**µ**L**		0.006		0.001
<50	1		1	
50–99	0.93 (0.83–1.03)		0.93 (0.83–1.04)	
100 – 199	0.86 (0.78–0.94)		0.83 (0.75–0.91)	
≥200	0.89 (0.80–0.99)		0.87 (0.78–0.98)	

Note: Age was centered; and missing values for BMI, clinical stage, and CD4 cell counts were included as a separate category. Adjusted incidence rate ratios [aIRR] of all-cause toxicity for the interaction between duration of exposure and stavudine dosage were 1.28 (95% CI 1.10–1.49), 0.66 (95% CI 0.49–0.89), and 0.69 (95% CI 0.32–1.51) for the second, third, and fourth years of exposure to stavudine 40 mg, respectively, compared to the first year of stavudine 30 mg. The corresponding aIRR of specific toxicity were 1.14 (95% CI 0.97–1.34), 0.56 (95% CI 0.40–0.79), and 0.47 (95% CI 0.17–1.29), respectively. P value from likelihood rate ratio tests for association were calculated across all categories of a given variable.

Women (aRR 1.18, 95%CI 1.11–1.26) and patients of older age (aRR 1.05, 95%CI 1.04–1.05 per year increase) had increased toxicity ratios. Advanced clinical stage (aRR 1.12, 95%CI 1.03–1.23 for stage 4; compared to stage 1 or 2), presence of tuberculosis at ART start (aRR 1.15, 95%CI 1.04–1.26), and low initial CD4 cell counts (aRR 0.93, 95%CI 0.83–1.03 for 50–99; aRR 0.86, 95%CI 0.78–0.94 for 100–199; and aRR 0.89, 95%CI 0.80–0.99 for ≥200; compared to <50 cells/µl) were also associated with increased toxicity ratios. Estimates for specific stavudine toxicity were similar to those reported for all-cause toxicity, although the association with specific toxicity was stronger for period of exposure (Table 3). Results from analyses restricted to the subgroup of patients with complete CD4 cell count, clinical stage, and BMI data did not differ from those reported above (Table S2).

### Timing of Neuropathy and Lipodystrophy Diagnosis

During the first 4 years of ART use, a total of 3,803 patients were diagnosed with peripheral neuropathy and 321 with lipodystrophy. Rates of lipodystrophy increased over time while rates of polyneuropathy where highest early after ART start and then decreased over time (Figure S3). Timing of lipodystrophy diagnosis was 12% shorter in patients receiving stavudine 40 mg than in those treated with 30 mg (*P* = 0.03). Although the size of the effect observed for polyneuropathy was similar (time reduction of 13%), this association was of borderline significance (*P* = 0.07). Sensitivity analyses including toxicity events diagnosed within 3 months of ART start gave similar results, showing shorter timing of lipodystrophy (12%, *P* = 0.03) and polyneuropathy (11%, *P* = 0.01) associated with the use of high stavudine dose.

## Discussion

In this large multicentric analysis of data from HIV-infected adults treated with stavudine-containing first-line regimens in resource-limited settings, we observed rates of all-cause toxicity of 7.80 per 100 person-years, but with large variation across cohorts. The majority of toxicities reported were events commonly associated with stavudine use. During the first and second years of ART use, patients treated with stavudine 40 mg had rates of toxicity 1.18 and 1.51 times higher, respectively, than those who received the 30 mg dose. Nevertheless, this relationship was reversed in subsequent years of treatment. Timing of toxicity diagnosis was approximately 12% shorter in patients who received the higher stavudine dose.

Previous longitudinal studies have reported 1-year Kaplan-Meier estimates of incident peripheral neuropathy ranging from 10% to 36% of patients having started ART [16], [17], and showed higher percentages of diagnoses among patients with AIDS. Lipodystrophy has also been frequently diagnosed in Indian patients treated with stavudine for a median of 20 months (46% had lipodystrophy and 27% lipoatrophy) [18]. Among HIV-infected patients in Senegal, prevalence of lipodystrophy was 40% for patients who had received stavudine for less than 3 years [19]. Overall estimates of toxicity incidence observed in our analysis fall within the ranges of rates reported in previous studies, but large differences in estimations were seen across sites. It is however likely that we underestimated rates of toxicity because of underreporting of less severe cases, especially of lipodystrophy and lactic acidosis, and because complete neurological examination is not routinely done in the programs. Subclinical cases of peripheral neuropathy and those diagnosed in patients who did not complain of symptoms are therefore likely to have been excluded from the estimations.

Rates of toxicity associated with the two drug doses were higher during the second than during the first year of therapy use, but they decreased with longer time of follow-up, resulting in an artificial protective effect among patients with long-term stavudine exposure. Other studies have reported similar effects of drug exposure for peripheral neuropathy [13], [14], probably reflecting patients who are more susceptible to the development of toxicity, especially neuropathy, and who would develop symptoms rapidly after the start of drug exposure.

During the first year of ART use, all-cause toxicity rate among patients using stavudine 40 mg was 1.18 times higher than among those treated with 30 mg, and estimates were similar when specific stavudine toxicity was studied. Adjusted incidence rate ratios increased with time of drug exposure over the first 2 years of ART use, was lower during the third year and similar during the fourth year. This finding together with the observation of a shorter delay in toxicity diagnosis associated with use of regimens containing stavudine 40 mg provides evidence of the cumulative dose effect of this drug.

Findings from safety studies including small numbers of patients have reported dose-related increases in peripheral neuropathy [1], [2], [4], treatment discontinuation [5], [20], reduction in body mass fat and malar fat thickness [20], and lipoatrophy [3] associated with a higher use of stavudine dose. In an open-label, 48-week study among patients treated with stavudine 40 mg for a median of 6 years, dose reduction to 30 mg led to improvement in symptomatic neuropathy in 23% of patients and to a reduction in lactate levels [21]. In contrast, a similar dose reduction or substitution of stavudine by tenofovir had minor or no effect on mitochondrial function in peripheral mononuclear cells in a study [22]. In another study the increase in mtDNA content in peripheral blood mononuclear of patients was not associated with an improvement in lipoatrophy after 1 year of follow-up [23]. Patients with long exposure to stavudine might therefore never completely recover or require long time periods to see improvements in adverse events.

The increased rates of toxicity observed in women, patients of older age, and those with advanced clinical disease or active tuberculosis observed in our analysis are consistent with findings reported from clinical studies. In a longitudinal study conducted in Canada, women had increased risk of and shorter time to lactic acidosis diagnosis than men, but no difference was observed in treatment-limiting lipodystrophy [24]. Other studies did not report differences in prevalent [13] or incident peripheral neuropathy [17] in men and women, but most previous studies include very few numbers of women. The difference in risk of toxicity observed in men and women could relate to differences in susceptibility or to a higher level of adherence to therapy achieved by women and not fully captured by the proxy adherence indicator based on missed clinical appointments.

Higher risk of peripheral polyneuropathy in older patients has also been reported in both pre-ART [25] and ART studies [7], [13], [14], [26] and could be related to longer exposure to HIV and/or ARV drugs [27]. The increased risk of toxicity observed with low CD4 cell counts has also been reported in studies conducted before [25], [28] and after the start of ART [13]–[15]. Prevalence [15], [16] and incidence [16], [25] of peripheral neuropathy have also been reported to be higher in patients with AIDS than among non-AIDS patients; shorter time to diagnosis has been observed in patients with history of AIDS diagnosis [17]; and higher risk of progression of established distal symmetric polyneuropathy, measured as an increase of the total neuropathy score, has been associated with reduction in CD4 levels [29]. The identification of advanced disease as a risk factor for toxicity could be explained by misclassification of patients with primary HIV-associated neuropathy as ART toxic neuropathy. These two forms of sensory peripheral neuropathy are the most frequent neurological disorders affecting HIV-infected patients [6]. Nevertheless, because the main risk factor analysis only included patients who were receiving stavudine-based therapy for at least 3 months and episodes diagnosed after this time, patients with clinically significant neuropathy diagnosed before would have not been included in the calculation of toxicity rates. It is therefore reasonable to attribute the observed toxic effect to stavudine. This observation is consistent with findings from recent *in vitro* studies that identified a threshold of 80% reduction of mtDNA through mutation or depletion mechanism before tissue pathology develops [30], [31] and with a trial investigating the effect of reducing the dose of stavudine on metabolic parameters and reporting a correlation between mtDNA and CD4 count levels [32]. It is also in agreement with researchers who hypothesized that patients diagnosed with severe immunodeficiency would be more susceptible to axonal injury and development of subsequent distal sensitive neuropathy [27]. Furthermore, the observed increased risk of toxicity in patients who had active tuberculosis at the start of ART could be explained by concomitant use of isoniazid and/or because of the severity of HIV disease of these patients.

Residual confounding due to absence of information about concomitant pathologies such as diabetes mellitus [13], [14], vitamin B12 deficiency, or alcoholism [32], or to treatment with other neurotoxic drugs might partly affect the estimations made in our risk factor analysis. Nevertheless, these factors are unlikely to have had an important impact since major known confounding factors were considered. Furthermore, results from sensitivity analyses including only patients with complete clinical stage, CD4 count, and BMI data did not differ from those presented in the main analysis.

This study was based on analysis of routine monitoring data collected in several HIV programs. Although this study was observational and dose allocation to patients was not random, dosage was based on changes in WHO recommendations rather than individualized patient management. Before 2009, patients of low body weight were the patients who received 30 mg stavudine-containing regimens. After 2009, the two drug dosages were available for prescription in the programs until the stocks of stavudine 40 mg stored in the pharmacies were finished. Patients diagnosed with minor stavudine-related toxicity were the first ones to be changed from 40 to 30 mg dose. Furthermore, the risk factor analysis and comparison of the timing of toxicity diagnosis were adjusted for major confounding factors reported in the literature. Also, clinicians working in the programs were unaware that this evaluation would be conducted. Because of the relatively long length of study follow-up (4 years), the patterns of reporting toxicity likely changed over time, based on the level of experience and type of provider. This might have led to underestimation of the toxicity ratios comparing the two dose groups, given that high stavudine dose was used in earlier calendar years. To account for these effects, reported estimates were also adjusted for both period of toxicity diagnosis and year of exposure.

In conclusion, in this study conducted in severely immunosuppressed patients, incidence of adverse events associated with the use of stavudine was high, particularly during the first two years of drug exposure. Although the use of high dose of stavudine (40 mg) was associated with a higher rate of toxicity and shorter delay in toxicity diagnosis, these findings highlight the need to use alternative, better tolerated nucleoside reverse transcriptase inhibitor drugs as recommended in current WHO guidelines.

## Supporting Information

Figure S1 **CONSORT diagram displaying cohort selection and inclusion in the analysis.** Figure note: MSF cohorts active in January 2010; LTFU, lost to follow-up.
(DOC)

Figure S2 **Kaplan-Meier estimates of the cumulative probability of A) all-cause and B) specific stavudine toxicity, stratified by stavudine dose group.** Figure note: The shaded area represents 95% confidence intervals for Kaplan-Meier estimates.
(TIFF)

Figure S3 **Rates of A) polyneuropathy and B) lipodystrophy, stratified by stavudine dose group.** Figure note: The lines represent predicted rates from the accelerated time failure models. Rates for each type of toxicity were modeled with an overall intercept (constant) representing the patient group with reference values for each of the variables included in the model: rural site, men, age centred at 0 years, toxicity diagnosis in 2005, initial clinical stage 1 or 2, initial BMI of <16 kg/m^2^, initial CD4 cell count of <50 cells/µL, and absence of tuberculosis treatment at ART start.
(TIFF)

Table S1 **Characteristics of ART sites, numbers of patients, and duration of follow-up. **Table note: IQR, interquartile range.
(DOC)

Table S2 **Associations between toxicity and individual level factors among patients with complete initial clinical stage, body mass index, and CD4 cell count data. **Table note: aIRR, adjusted incidence rate ratios from multivariable mixed-effect Poisson models; BMI, body mass index; CI, confidence interval; *P* value from likelihood rate ratio tests for association calculated across categories of given variable.
(DOC)
