# Supplementary material for: Toxicity Associated with Stavudine Dose Reduction from 40 to 30 mg in First-Line Antiretroviral Therapy
Source: PLoS One. 2011 Nov 21;6(11):e28112. doi: 10.1371/journal.pone.0028112 (PMC3221698; doi:10.1371/journal.pone.0028112)
Supplement: Figure S1 — CONSORT diagram displaying cohort selection and inclusion in the analysis. Figure note: MSF cohorts active in January 2010; LTFU, lost to follow-up. (DOC) [file pone.0028112.s001.doc]

MSF cohorts (n=64)

Cohorts assessed for eligibility (n=49)

No FUCHIA electronic monitoring (n=15)

Cohorts included (n=23)

Excluded (n=26)

Reasons for exclusion: ≥20% LTFU after 1 year of ART start (n=1)

<50 patients meeting study criteria (n=25)

Figure note: MSF cohorts active in January 2010; LTFU, lost to follow-up
