# Supplementary figures and images for: Toxicity Associated with Stavudine Dose Reduction from 40 to 30 mg in First-Line Antiretroviral Therapy
Source: PLoS One. 2011 Nov 21;6(11):e28112. doi: 10.1371/journal.pone.0028112 (PMC3221698; doi:10.1371/journal.pone.0028112)

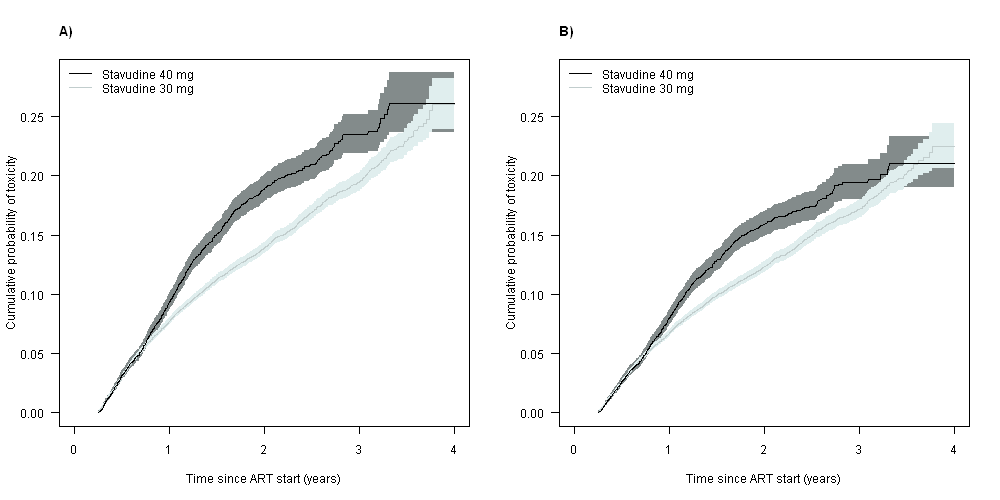

Supplement: Figure S2 — Kaplan-Meier estimates of the cumulative probability of A) all-cause and B) specific stavudine toxicity, stratified by stavudine dose group. Figure note: The shaded area represents 95% confidence intervals for Kaplan-Meier estimates. (TIFF) [file pone.0028112.s002.tiff]

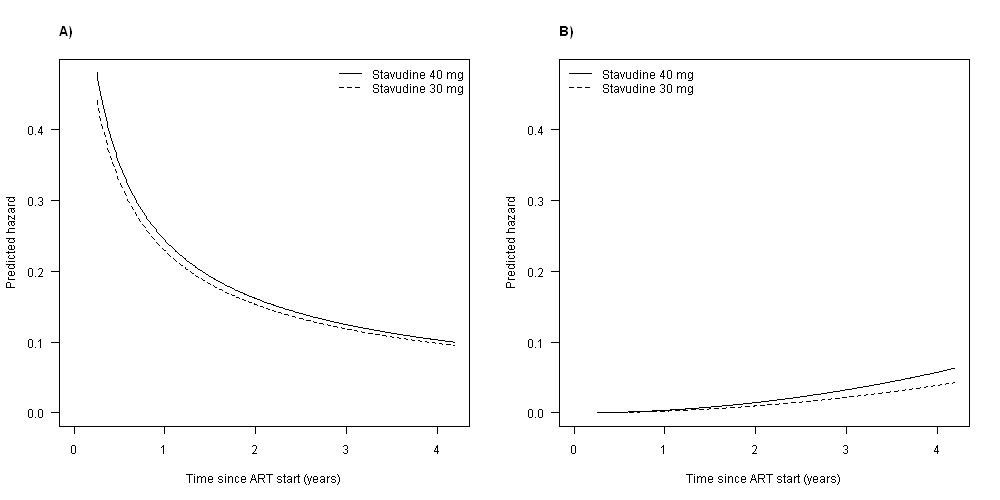

Supplement: Figure S3 — Rates of A) polyneuropathy and B) lipodystrophy, stratified by stavudine dose group. Figure note: The lines represent predicted rates from the accelerated time failure models. Rates for each type of toxicity were modeled with an overall intercept (constant) representing the patient group with reference values for each of the variables included in the model: rural site, men, age centred at 0 years, toxicity diagnosis in 2005, initial clinical stage 1 or 2, initial BMI of <16 kg/m2, initial CD4 cell count of <50 cells/µL, and absence of tuberculosis treatment at ART start. (TIFF) [file pone.0028112.s003.tiff]
