# Supplementary material for: Toxicity Associated with Stavudine Dose Reduction from 40 to 30 mg in First-Line Antiretroviral Therapy
Source: PLoS One. 2011 Nov 21;6(11):e28112. doi: 10.1371/journal.pone.0028112 (PMC3221698; doi:10.1371/journal.pone.0028112)
Supplement: Table S1 — Characteristics of ART sites, numbers of patients, and duration of follow-up. Table note: IQR, interquartile range. (DOC) [file pone.0028112.s004.doc]

**Table S1. Characteristics of ART sites, numbers of patients,** and duration of follow-up

| **Country** | **No. of sites** | **Type of setting** | **Year of ART start** | **Stavudine 40 mg** | | | **Stavudine 30 mg** | | |
| --- | --- | --- | --- | --- | --- | --- | --- | --- | --- |
|  |  |  |  | **No. of patients** | **Median months of follow-up [IQR]** | **Median cumulative stavudine exposure (mg)** | **No. of patients** | **Median months of follow-up [IQR]** | **Median cumulative stavudine exposure (mg)** |
| Burkina Faso | 1 | Urban | 2003 | 450 | 20.7 [13.3 - 29.0] | 49,715 [32,013 - 69,546] | 2022 | 14.3 [7.4 - 24.4] | 25,666 [13,365 - 43,880] |
| Cameroon | 2 | Urban | 2003-04 | 893 | 16.0 [9.3 - 24.4] | 38,321 [22,393 - 58,507] | 1413 | 11.8 [7.0 – 79.4] | 21,230 [12,537 - 34,950] |
| Democratic Republic of Congo | 1 | Rural | 2003 | 79 | 13.9 [8.1 - 26.1] | 33,354 [19,555 - 62,528] | 642 | 15.9 [8.7 - 26.9] | 28,652 [15,612 - 48,493] |
| Republic of Guinea | 2 | Urban | 2003-04 | 298 | 11.4 [7.0 – 18.8] | 27,361 [16,716 - 45,102] | 2722 | 11.1 [6.4 - 17.8] | 19,929 [11,532 - 31,993] |
| Nigeria | 1 | Urban | 2004 | 418 | 16.7 [11.0 - 22.2] | 39,977 [26,494 - 53,303] | 719 | 13.8 [7.5 - 20.3] | 24,897 [13,543 - 36,606] |
| Kenya | 4 | Rural & urban | 2001-04 | 1110 | 20.5 [11.6 – 29.0] | 49,084 [27,755 - 69,546] | 4106 | 15.3 [7.5 – 26.5] | 27,588 [13,543 - 47,724] |
| Uganda | 1 | Rural | 2002 | 404 | 10.4 [6.6 - 16.0] | 25,074 [15,928 - 38,400] | 2267 | 16.5 [8.9 - 26.4] | 29,687 [15,967 - 47,488] |
| Malawi | 1 | Rural | 2001 | 554 | 12.7 [6.9 - 24.3] | 30,397 [16,559 - 58,349] | 7226 | 13.1 [7.0 - 21.9] | 23,655 [12,596 - 39,386] |
| Mozambique | 5 | Urban | 2002-05 | 1680 | 17.0 [10.6 – 25.2] | 40,844 [25,469 - 60,399] | 7665 | 13.0 [7.7 – 20.7] | 23,478 [13,897 - 37,257] |
| Zimbabwe | 4 | Urban | 2004-06 | 1872 | 16.0 [10.4 – 22.3] | 38,439 [25,074 - 53,460] | 11,141 | 13.3 [7.1 – 21.0] | 23,951 [12,715 - 37,730] |
| Cambodia | 1 | Urban | 2003 | 55 | 11.3 [4.8 - 15.6] | 27,203 [11,512 – 37,375] | 1049 | 12.9 [8.9 - 17.7] | 23,241 [16,026 - 31,816] |
| ***TOTAL*** | 23 |  |  | 7813 | 16.2 [9.7 - 24.2] | 38,952 [23,340 – 58,191] | 40,972 | 13.4 [7.3 - 21.8] | 24,069 [13,247 - 38,972] |
| ***Total Sub-Saharan Africa*** | 22 |  |  | 7758 | 16.2 [9.7 - 24.2] | 39,110 [23,340 – 58,349] | 39,923 | 13.4 [7.4 - 21.7] | 24,069 [13,188 - 39,327] |

Note: IQR, interquartile range
