# Supplementary material for: Toxicity Associated with Stavudine Dose Reduction from 40 to 30 mg in First-Line Antiretroviral Therapy
Source: PLoS One. 2011 Nov 21;6(11):e28112. doi: 10.1371/journal.pone.0028112 (PMC3221698; doi:10.1371/journal.pone.0028112)
Supplement: Table S2 — Associations between toxicity and individual level factors among patients with complete initial clinical stage, body mass index, and CD4 cell count data. Table note: aIRR, adjusted incidence rate ratios from multivariable mixed-effect Poisson models; BMI, body mass index; CI, confidence interval; P value from likelihood rate ratio tests for association calculated across categories of given variable. (DOC) [file pone.0028112.s005.doc]

**Table S2. Associations between toxicity and individual level factors among patients with complete initial clinical stage, body mass index, and CD4 cell count data**

|  | **All-cause toxicity** | | **Typical stavudine toxicity** | |
| --- | --- | --- | --- | --- |
| N=32,444 | **aIRR (95% CI)** | ***P* value** | **aIRR (95% CI)** | ***P* value** |
| **Stavudine dose** |  | <0.001 |  | <0.001 |
| 30 mg | 1 |  | 1 |  |
| 40 mg | 1.20 (1.06-1.36) |  | 1.18 (1.04-1.36) |  |
| **Time of drug exposure (year)** |  | 0.007 |  | 0.009 |
| 1 | 1 |  | 1 |  |
| 2 | 0.98 (0.90-1.08) |  | 1.00 (0.91-1.10) |  |
| 3 | 0.96 (0.82-1.12) |  | 0.94 (0.79-1.11) |  |
| 4 | 1.14 (0.83-1.57) |  | 1.07 (0.75-1.52) |  |
| **Age, per 1 year increase** | 1.04 (1.04-1.05) | <0.001 | 1.05 (1.04-1.05) | <0.001 |
| **Gender** |  | <0.001 |  | <0.001 |
| Men | 1 |  | 1 |  |
| Women | 1.19 (1.09-1.29) |  | 1.16 (1.07-1.27) |  |
| **Period of toxicity diagnosis** |  | <0.001 |  | <0.001 |
| 2005 | 1 |  | 1 |  |
| 2006 | 2.17 (1.76-2.67) |  | 2.43 (1.92-3.06) |  |
| 2007 | 2.51 (2.05-3.08) |  | 2.96 (2.36-3.73) |  |
| 2008 | 2.19 (1.78-2.70) |  | 2.47 (1.96-3.13) |  |
| 2009 | 6.53 (4.46-9.55) |  | 6.92 (4.41-10.85) |  |
| **Clinical WHO stage** |  | 0.088 |  | 0.425 |
| 1/2 | 1 |  | 1 |  |
| 3 | 1.06 (0.97-1.16) |  | 1.05 (0.96-1.15) |  |
| 4 | 1.13 (1.01-1.26) |  | 1.08 (0.95-1.21) |  |
| **Tuberculosis diagnosis at ART start** |  | 0.115 |  | 0.184 |
| No | 1 |  | 1 |  |
| Yes | 1.10 (0.98-1.24) |  | 1.09 (0.96-1.24) |  |
| **BMI group, kg/m^2^** |  | <0.001 |  | <0.001 |
| <16 | 1 |  | 1 |  |
| 16-18.49 | 0.93 (0.81-1.08) |  | 0.98 (0.82-1.14) |  |
| 18.50-24.99 | 0.86 (0.75-0.99) |  | 0.89 (0.76-1.04) |  |
| ≥25 | 1.10 (0.92-1.32) |  | 1.20 (0.98-1.46) |  |
| **CD4 cell count, cells/µL** |  | 0.104 |  | 0.014 |
| <50 | 1 |  | 1 |  |
| 50-99 | 0.96 (0.86-1.08) |  | 0.95 (0.84-1.07) |  |
| 100-199 | 0.89 (0.80-0.98) |  | 0.85 (0.76-0.94) |  |
| ≥200 | 0.93 (0.82-1.04) |  | 0.90 (0.79-1.01) |  |

Note Table S2. aIRR, adjusted incidence rate ratios from multivariable mixed-effect Poisson models; BMI, body mass index; CI, confidence interval; *P* value from likelihood rate ratio tests for association calculated across categories of given variable.
